# Endpoint Visual Detection of Three Genetically Modified Rice Events by Loop-Mediated Isothermal Amplification

**DOI:** 10.3390/ijms131114421

**Published:** 2012-11-07

**Authors:** Xiaoyun Chen, Xiaofu Wang, Nuo Jin, Yu Zhou, Sainan Huang, Qingmei Miao, Qing Zhu, Junfeng Xu

**Affiliations:** 1 Institute of Agriculture Quality and Standard for Agro-products, Zhejiang Academy of Agricultural Sciences, Hangzhou 310021, China; E-Mails: yxchen77@sina.com (X.C.); yywxf1981@yahoo.cn (X.W.); microbes@yahoo.cn (Y.Z.); hsn871015@163.com (S.H.); tracymiao2006@yahoo.com.cn (Q.M.); zhuqing851006@yahoo.cn (Q.Z.); 2 School of Biotechnology, East China University of Science and Technology, Shanghai 200237, China; E-Mail: jinnuo2012@126.com; 3 College of Chemistry and Life Science, Shenyang Normal University, Shenyang 110034, China

**Keywords:** GMO detection, LAMP, transgenic rice, TT51-1, KMD1, KF6

## Abstract

Genetically modified (GM) rice KMD1, TT51-1, and KF6 are three of the most well known transgenic Bt rice lines in China. A rapid and sensitive molecular assay for risk assessment of GM rice is needed. Polymerase chain reaction (PCR), currently the most common method for detecting genetically modified organisms, requires temperature cycling and relatively complex procedures. Here we developed a visual and rapid loop-mediated isothermal amplification (LAMP) method to amplify three GM rice event-specific junction sequences. Target DNA was amplified and visualized by two indicators (SYBR green or hydroxy naphthol blue [HNB]) within 60 min at an isothermal temperature of 63 °C. Different kinds of plants were selected to ensure the specificity of detection and the results of the non-target samples were negative, indicating that the primer sets for the three GM rice varieties had good levels of specificity. The sensitivity of LAMP, with detection limits at low concentration levels (0.01%–0.005% GM), was 10- to 100-fold greater than that of conventional PCR. Additionally, the LAMP assay coupled with an indicator (SYBR green or HNB) facilitated analysis. These findings revealed that the rapid detection method was suitable as a simple field-based test to determine the status of GM crops.

## 1. Introduction

The production of genetically modified (GM) crops is increasing annually worldwide. By 2011, genetically modified (GM) crops were being grown by 16.7 million farmers distributed across 29 countries and 160 million hectares [1]. In rice, GM technology has been used to confer herbicide tolerance and pathogen or insect resistance characteristics [2–6]. Rice is a leading crop in China, and substantial efforts have been devoted to its transgenesis. At least 100 transgenic lines have been developed over the past 10 years [7–10]. At present, GM rice KMD1, TT51-1, and KF6 are three of the most well known events [11–16], especially TT51-1, which obtained biosafety certificates from the Chinese government in 2009 [17]. With global expansion in the areas sown to transgenic crops, the likelihood of contamination of non-transgenic varieties with GM products is increasing. Examples include the accidental presence of herbicide tolerant rice in Europe, and TT51-1 rice on the Chinese market [18,19]. To monitor the presence of GM ingredients, methods of GM detection that allow for proper labeling are now required in over 30 countries [20].

Polymerase chain reaction (PCR) or real-time PCR is currently the most sensitive and specific method of GM detection. Real-time PCR assays for the rapid detection of KMD1, TT51-1, and KF6 have also been recently reported [21–23]. The PCR method, however, requires expensive equipment and trained personnel for its implementation. In addition, *Taq* DNA polymerase in PCR assays can be inactivated by inhibitors present in crude biological samples [24] and thus may not be applicable for field trials of GM detection. Thus, another rapid, simple, and effective assay is needed to supplement the current PCR methods.

Loop-mediated isothermal amplification (LAMP), originally developed by Notomi *et al.*[25], is a very sensitive, easy, and timesaving method. The LAMP assay relies on a set of four specially designed primers, inner primers (FIP and BIP) and outer primers (F3 and B3), that can recognize at least six independent target DNA sequences to reduce nonspecific binding, thus ensuring its specificity. A series of DNA fragments containing multiple units of the target sequence were obtained under isothermal conditions (60–65 °C) utilizing the displacement properties of the Bst DNA Polymerase Large Fragment (New England Biolabs, Sumida, Tokyo, Japan). A simple incubator, such as a water bath or heating block, is sufficient for DNA amplification, making this method feasible for use under field conditions. In addition, LAMP can yield 10^9^ copies of the amplification product from one molecule of the starting material [25]. Moreover, the LAMP amplification products tend to be far longer than conventional PCR products and thus easier to detect via end-point analysis. The LAMP technique is widely used to detect bacterial microorganisms [26,27], viruses [28,29], and parasites [30,31]. Few applications for genetically modified organism (GMO) detection using the LAMP technique, however, have been reported [32–35]. In the present study, we designed corresponding primers and optimized visual (adding SYBR green or hydroxy naphthol blue [HNB]) LAMP methods for detection of the GM rice events KMD1, TT51-1, and KF6. The specificity and sensitivity of the primers in the LAMP reactions were determined. The results indicated that the developed LAMP assays were more specific and sensitive than conventional PCR assays and could be used for GMO detection in the field.

## 2. Results and Discussion

### 2.1. Primer Design for the Target Sequences

**Figure 1:**
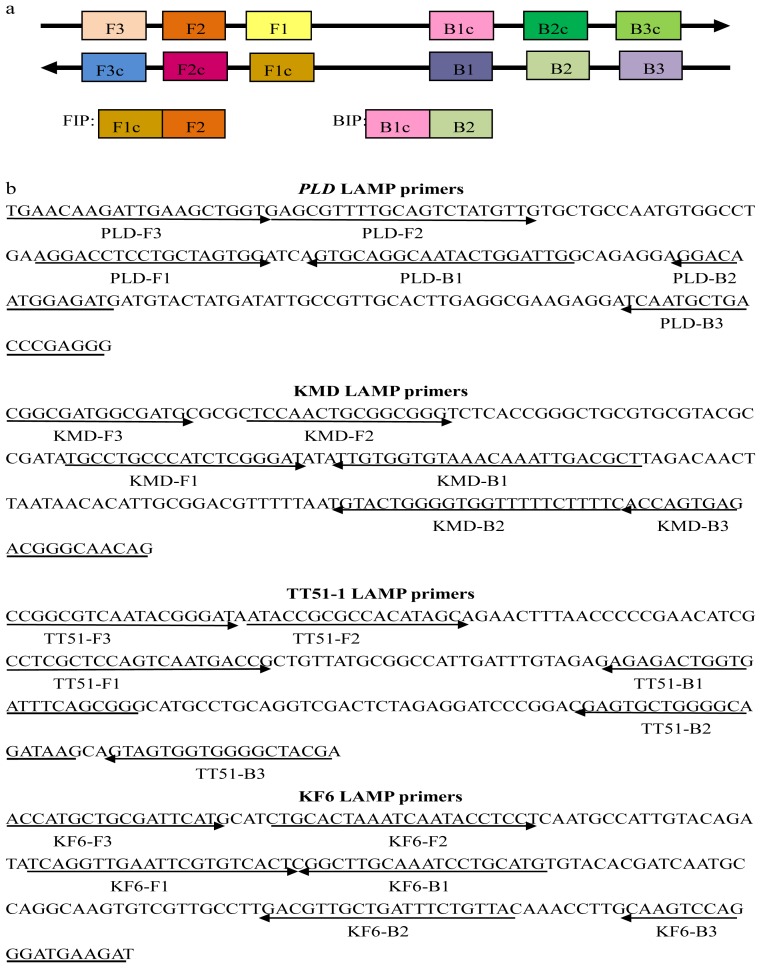
Primer design for loop-mediated isothermal amplification (LAMP) assays. (**a**) Schematic diagram of LAMP primer design; (**b**) Nucleotide sequences used for designing the primers. Primers used for the LAMP assay are indicated by the arrows.

The LAMP method was performed using four specially designed primers that recognized a total of six distinct sequences on the target DNA to specifically amplify the target sequences. The design of the LAMP primers was based on the six regions in the target sequence, designated in Figure 1a from the origination-end as F3, F2, F1, B1, B2, and B3. The Forward Inner Primer (FIP) consisted of the F2 sequence (at its 3′ end), which is complementary to the F2c region, and the same sequence as that of the F1c region at its 5′ end. For specific detection of the three transgenic rice events, junction sequences of the three GM rice events were collected as the target sequence. KMD1 LAMP primers were designed according to the 188-bp event-specific sequence of the 3′ end sequence of exogenous integration [21]. The TT51-1 primers were designed based on the 3′ junction [22] of this event and the target sequence was 202 bp. LAMP primers for KF6 were designed based on the 5′ junction [23] to recognize a 187-bp target sequence. The gene encoding phospholipase D (*PLD*) was selected for rice genome control [36]. The *PLD* LAMP primers were targeted to a 183-bp sequence of the gene exon (Genbank number AB001919, 3758-3941bp). The primers used in this research are listed in Table 1 and detailed locations of LAMP primers in the target DNA sequences are shown in Figure 1b.

### 2.2. Optimization of the LAMP Reactions

To visually detect the GM rice events, SYBR green and HNB were employed to evaluate the results of the LAMP assay. We first tested the efficiencies of LAMP by adding SYBR green or HNB before and after the reaction (data not given). The LAMP-amplified products could be directly observed by the naked eye by adding 1.5 μL 1000× SYBR green I to the reaction mixture. A positive LAMP reaction, *i.e.*, the color of reaction mixture turned green while the mixture remained orange, indicated negative or no amplification.

The LAMP reaction produced large amounts of the insoluble product magnesium pyrophosphate, which was generated by pyrophosphate ions (a byproduct in LAMP) and Mg^2+^ ions. As the Mg^2+^ ion concentration decreases with the progression of the LAMP reaction, the LAMP reaction can be quantified by measuring the Mg^2+^ ion concentration in the reaction solution. Based on this phenomenon, Goto *et al.*[37] reported a simple colorimetric assay for detection of the LAMP reaction by adding HNB, a metal indicator, to the pre-reaction solution. According to their report, the optimal final concentration of HNB in the reaction solution is 120 μM. The color change of the LAMP reaction mixture with HNB from violet to sky blue indicates a positive reaction. In addition, different primer concentrations and the ratios between the inner primers (FIP and BIP) and outer primers (F3 and B3) were also optimized. The optimized LAMP reaction conditions are described in the “Experimental Section”.

**Figure 2:**
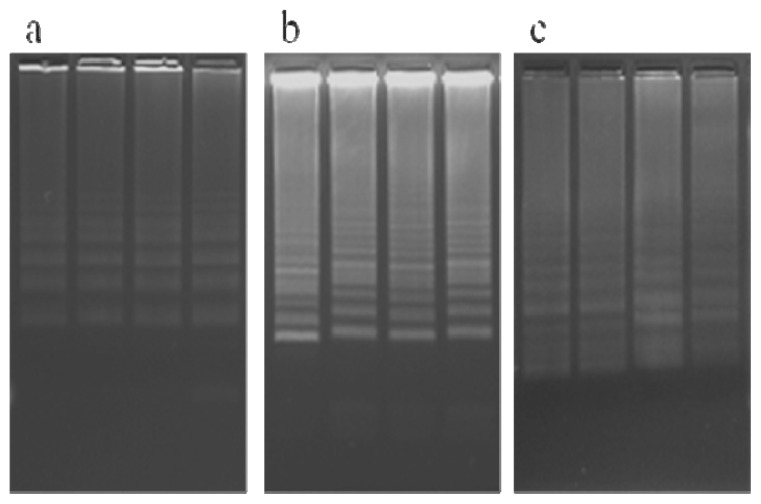
Determination of the optimal reaction temperature for the KMD1 LAMP assays. LAMP products were detected on 2% agarose gel after 60-min amplification with 5 ng of the KMD1 DNA template at 60 °C (**a**), 63 °C (**b**), and 65 °C (**c**). The result of each reaction was verified by four replications.

To test the optimal reaction temperature of the developed LAMP assays, different reaction temperatures (60 °C, 63 °C, and 65 °C) were selected for the KMD1, TT51-1, and KF6 LAMP assays. As shown in Figure 2, the KMD1 event-specific LAMP products could be observed after 60-min amplification with 5 ng DNA template at 60 °C, 63 °C, and 65 °C. Similar results were obtained for the TT51-1 and KF6 event-specific LAMP assays (data not shown). The results indicated that the optimal reaction temperature for the established LAMP assays was 63 °C.

### 2.3. Specificity of LAMP

**Figure 3:**
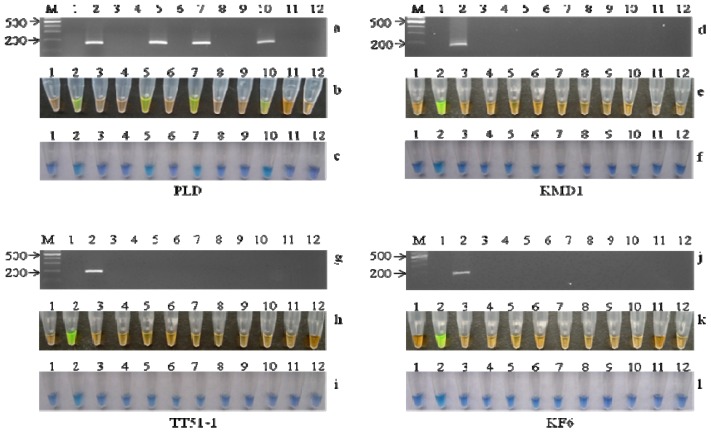
Specificity of conventional polymerase chain reaction (PCR) and LAMP assays using SYBR green and HNB. (**a**) Conventional PCR results of the phospholipase D (PLD) specificity test; (**b**) and (**c**) show the LAMP assay results of PLD specificity using SYBR green and HNB, respectively. Lane M, 1000 marker; Lane 1, no-template control (NTC); Lanes 2–12, non-GM rice, GTS40-3-2, MON531, KMD1, GT73, TT51-1, Mon863, *Arabidopsis thaliana*, KF6, non-GM pepper, and non-GM tomato; (**d**) Conventional PCR results of the KMD1 specificity test; (**e**) and (**f**) are LAMP assay results of KMD1 specificity using SYBR green and HNB, respectively. Lane M, 1000 marker; Lane 1, no-template control (NTC); Lanes 2–12, KMD1, non-GM rice, GTS40-3-2, MON531, GT73, TT51-1, Mon863, *A. thaliana*, KF6, non-GM pepper, and non-GM tomato; (**g**) conventional PCR results of the TT51-1 specificity test; (**h**) and (**i**) are LAMP assay results of TT51-1 specificity using SYBR green and HNB, respectively. Lane M, 1000 marker; Lane 1, no template control (NTC); Lanes 2–12, TT51-1, non-GM rice, GTS40-3-2, MON531, GT73, KMD1, Mon863, *A. thaliana*, KF6, non-GM pepper, and non-GM tomato; (**j**) conventional PCR results of the KF6 specificity test; (**k**) and (**l**) show the LAMP assay results of KF6 specificity using SYBR green and HNB, respectively. Lane M, 1000 marker; Lane 1, no template control (NTC); Lanes 2–12, KF6, non-GM rice, GTS40-3-2, MON531, GT73, KMD1, Mon863, *A. thaliana*, TT51-1, non-GM pepper, and non-GM tomato.

The specificities of the LAMP assays for detecting *PLD*, KMD1, TT51-1, and KF6 were confirmed by checking the reaction with various plant DNA samples. Several GM events (GTS40-3-2 soybean, Mon531 cotton, GT73 canola, and Mon 863 maize) and non-GM plants (rice, pepper, tomato and *Arabidopsis thaliana*) were used. In the specificity test, 50 ng of the total corresponding plant genomic DNA was used as a template in the LAMP and conventional PCR assays. In the *PLD* assay, a positive color of green or sky blue was obtained in the reaction mixtures using the rice genomic DNA as a template, and a negative color of orange or violet was observed in other reactions, as well as the no-template control (Figure 3b,c). In the PLD-F3/B3 amplicon derived from rice genomic DNA, 183-bp fragments were present, while all of the other templates failed to amplify from this primer pair (Figure 3a). The three GM rice events, KMD1, TT51-1 and KF6, were all specifically detected by the LAMP assay. The changed color was only observed in the LAMP mixture containing the corresponding GM rice event (Figure 3e,f,h,i,k,l). These results indicated that only the target DNA sequences were amplified and there was no cross-reaction between the three GM rice events and other crops. In addition, the results of the conventional PCR were consistent with those of the LAMP assay (Figure 3d,g,j). The products from the conventional PCR of *PLD*, KMD1, TT51-1, and KF6 were sequenced and confirmed the accuracy of the LAMP-amplified sequences. These data demonstrated that the established LAMP assays had high specificity for detecting the target DNAs.

### 2.4. Sensitivity of LAMP

**Figure 4:**
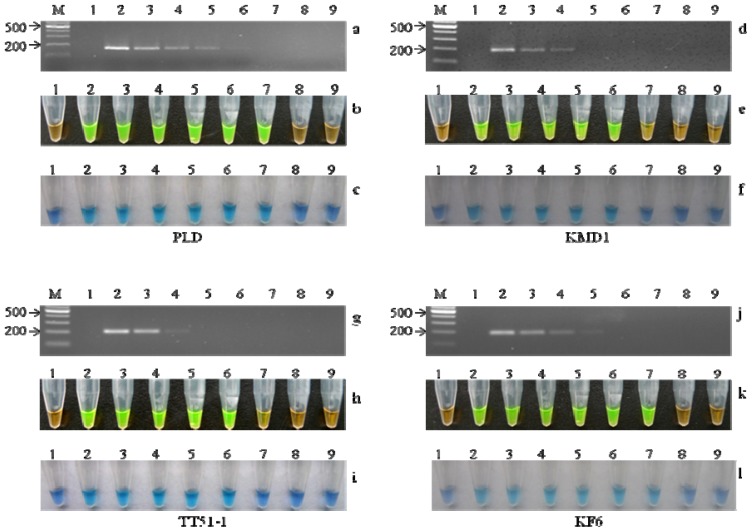
Sensitivity test of conventional PCR and visualization of the LAMP products using SYBR green and HNB. (**a**) Sensitivity test of PLD conventional PCR; (**b**) and (**c**) Sensitivity test of PLD and visualization of the LAMP products using SYBR green and HNB, respectively. Lane M, 1000 marker; Lane 1, NTC; Lanes 2–9, correspond to 100, 10, 1, 0.1, 0.01, 0.005, 0.001, and 0.0005 ng rice genomic DNA; (**d**) Sensitivity test of KMD1 conventional PCR; (**e**) and (**f**) Sensitivity test of KMD1 and visualization of the LAMP products using SYBR green and HNB, respectively. Lane M, 1000 marker; Lane 1, NTC; Lanes 2–9, mixed KMD1 samples with GM contents of 10%, 5%, 1%, 0.1%, 0.01%, 0.005%, 0.001%, and 0%; (**g**) Sensitivity test of TT51-1 conventional PCR; (**h**) and (**i**) Sensitivity test of TT51-1 LAMP products visualized using SYBR green and HNB, respectively. Lane M, 1000 marker; Lane 1, NTC; Lanes 2–9, mixed TT51-1 samples with GM contents of 10%, 5%, 1%, 0.1%, 0.01%, 0.005%, 0.001%, and 0%; (**j**) Sensitivity test of KF6 conventional PCR; (**k**) and (**l**) Sensitivity test of KF6 LAMP products visual observation using SYBR green and HNB, respectively. Lane M, 1000 marker; Lane 1, NTC; Lanes 2–9, mixed KF6 samples with GM contents of 10%, 5%, 1%, 0.1%, 0.01%, 0.005%, 0.001%, and 0%.

The sensitivity of this assay for *PLD* and each GM rice event was evaluated. To determine the sensitivity of the *PLD* LAMP assay, non-GM rice genomic DNA was serially diluted to final concentrations of 50, 5, 0.5, 0.05, 0.005, 0.0025, 0.0005 and 0.00025 ng/μL. Diluted DNA sample (2 μL) was used as a template in each reaction. As shown in Figure 4b,c, the detection limit of the PLD LAMP assay was 0.005 ng. The haploid genome size of rice was estimated to be 430 Mbp [38], corresponding to a weight of 0.47 pg. Therefore, the detection limit of the PLD LAMP assay was approximately 10 copies. For comparison purposes, conventional PCR was performed using PLD-F3 and PLD-B3 primers with the same amount of genomic DNA, and the detection limit of conventional PCR was 0.01 ng and approximately 20 copies (Figure 4a). It is likely that the sensitivity of the LAMP ‘endpoint’ detection supersedes that of ‘endpoint’ PCR detection (in terms of sensitivity), because far more LAMP-amplified products are generated during the reaction process compared to PCR.

To test the sensitivities of the KMD-1, TT51, and KF6 LAMP assays, the tested samples were prepared by adding the relevant GM rice DNA to non-GM rice DNA to produce nominal concentrations of 10%, 5%, 1%, 0.1%, 0.01%, 0.005%, 0.001%, and 0%. In each reaction, a total of 50 ng genomic DNA was amplified. As shown in Figure 4e,f,h,i, positive colors were observed for concentrations of 0.01% or above in both the KMD1 and TT51-1 LAMP assays. On the other hand, compared with the conventional PCR assays (Figure 4d,g), the sensitivity of the LAMP assays was 10 to 100 times higher. For the KF6 LAMP assay, our LAMP assay could detect levels at concentrations of 0.005% and the KF6 conventional PCR assay could detect levels at concentrations of 0.1% (Figure 4j–l). Therefore, the results of the LAMP assay complied with the definition of minimum performance requirements for analytical methods of GMO testing of the European network of GMO laboratories that the level of detection should be lower than 0.45% [39].

We next compared the results between visualization with added SYBR green/HNB and gel electrophoresis analysis, and found no difference (data not shown). This finding indicated that this visualization method could be used for LAMP analysis instead of general agarose gel electrophoresis. Moreover, without agarose gel electrophoresis of the LAMP assay, we could decrease the risk of contamination of other subsequent LAMP reactions as well as save time and labor. In addition, the performances of SYBR green and HNB in this study revealed specific advantages of each indicator. For HNB, the detection sensitivity was equivalent to that of the assay using SYBR green. In contrast, the brightness of HNB was significantly weaker than that of SYBR green. In the LAMP assay using HNB, however, it was unnecessary to open the tubes to determine whether the reaction was positive or negative and this reduced the risk of cross-contamination.

## 3. Experimental Section

### 3.1. Plant Materials

The samples of GM rice (KMD1, TT51-1, and KF6) and other plants with the transgenic events (GTS40-3-2 soybeans, Mon531 cotton, GT73 canola, and Mon863 maize) were kindly provided by the Center of Science and Technology Development, Ministry of Agriculture of the People’s Republic of China (Beijing, China). Non-transgenic plants of *Oryza sativa*, *A. thaliana*, *tomato*, and *pepper* were collected by our laboratory (Hangzhou, China).

### 3.2. DNA Extraction

For GM plants and non-transgenic rice, genomic DNA used in conventional PCR and LAMP was extracted and purified from seed flour using a kit (DNA Extraction Kit for GMO Detection v3.0, Takara, Shiga, Japan). For *A. thaliana*, *tomato* and *pepper*, genomic DNA was extracted and purified from young leaves with the same DNA Extraction Kit. The DNA present in the extracts was quantified by absorbance at 260 nm and further characterized by agarose gel electrophoresis.

### 3.3. Primer Design

The principles of primer design according to [25] were followed, and all of the LAMP primers were designed using the specific software of Primer Explorer V4 (Version v4; Fujitsu: Kobe, Japan) (http://primerexplorer.jp/elamp4.0.0/index.html) [40]. Each set of LAMP primers contained two inner primers (FIP and BIP) and two outer primers (F3 and B3). The scheme of the LAMP primer design for target sequences is shown in Figure 1a. All the primers used in this study were synthesized by Invitrogen Biotechnology Co., Ltd. (Shanghai, China) and are detailed in Table 1.

### 3.4. Conventional PCR

To compare the sensitivity and specificity of the established LAMP assays, conventional PCR was also performed. In our study, the sizes of the target sequences were all approximately 200 bp and thus the outer primers (F3 and B3) of each LAMP were suitable for use as forward and reverse primers in the corresponding conventional PCR system. Each conventional PCR mixture (30 μL volume) comprised 10 × PCR buffer (Takara Biotechnology Co.), 200 mM dNTP (Takara Biotechnology Co.), 0.5 mM of each primer, 1.25 U Taq DNA polymerase (Takara Biotechnology Co.), and template. The PCR regimen consisted of a 95 °C/3 min denaturation, followed by 35 cycles of 94 °C/30 s, 55 °C/30 s and 72 °C/30 s, with a final elongation step of 72 °C/7 min. The resulting amplicons were separated by electrophoresis through 2% agarose gels in 0.5× TBE with GelRed staining. The results of each PCR assay were verified by three replications and the assays were repeated three times.

### 3.5. LAMP Assay

The LAMP assay was performed in a 25 μL total reaction mixture containing 0.8 μM each FIP and BIP, 0.2 μM each F3 and B3, 400 μM each dNTP, and 1× ThermoPol Reaction Buffer. After adding the template DNA, the mixture was incubated for 5 min at 95 °C and cooled on ice, and 8 U of the large fragment of *Bst* DNA Polymerase (New England BioLabs) was added. The reaction mixture was incubated at 63 °C for 1 h and then heated at 80 °C for 5 min to terminate the reaction. The result of each LAMP assay was verified by three replications and the assays were repeated three times. In this study, HNB and SYBR green (Generay Biotech Co., Ltd., Shanghai, China) were used to indicate the results of the LAMP assay. For visualization of the LAMP reaction with SYBR green, 1.5 μL 1000 × SYBR green was added to each tube after the reaction. For visualization of the LAMP reaction with HNB, a final concentration of 120 μM HNB was added to the tube solution before reaction.

## 4. Conclusions

A visual and rapid detection method for GM rice KMD-1, TT51, and KF6 events using the LAMP technique was developed. Compared to classical PCR analysis, the LAMP assay has distinct advantages, such as efficiency, low cost, and simple procedures for analysis. The LAMP assay can be performed under optimized isothermal conditions (63 °C) without PCR thermal cyclers within a shorter period of time (60 min in this study). In combination with SYBR green or HNB, the amplified products can be directly visualized by the naked eye rather than conventional gel electrophoresis analysis, which contributes to reduce the risk of cross-contamination, typically a significant disadvantage of the LAMP assay [41]. In addition, the sensitivity of the developed KMD-1, TT51 and KF6 LAMP assays were more sensitive than conventional PCR methods. High specificity and sensitivity, cost-effectiveness, and low equipment requirements of the developed visual LAMP assays make this method more useful for GM rice sample analysis, especially as a field detection technique in locations where more complex laboratory equipment is not available.

## Figures and Tables

**Table 1:** Primers used in this research.

Primers name	Sequence (5′-3′)	Target	Amplicon size (bp)	Reference
PLD-F3	TGAACAAGATTGAAGCTGGTG	*PLD* gene	183	This work
PLD-B3	CCCTCGGGTCAGCATTGA			
PLD-FIP	TCCACTAGCAGGAGGTCCTTTTTAGCGTT TTGCAGTCTATGTTG			
PLD-BIP	GTGCAGGCAATACTGGATTGGTTTTCCT CTTCGCCTCAAGTGC			

KMD1-F3	CGGCGATGGCGATGC	Junction of KMD1	188	This work
KMD1-B3	CTGTTGCCCGTCTCACTGGT			
KMD1-FIP	TATCCCGAGATGGGCAGGCATTTTTCCA ACTGCGGCGGGT			
KMD1-BIP	TTGTGGTGTAAACAAATTGACGCTTTTTT GAAAAGAAAAACCACCCCAGTAC			

TT51-F3	CCGGCGTCAATACGGGATA	Junction of TT51-1	202	This work
TT51-B3	TCGTAGCCCCACCACTAC			
TT51-FIP	CGGTCATTGACTGGAGCGAGGTTTTATA CCGCGCCACATAGCA			
TT51-BIP	AGAGACTGGTGATTTCAGCGGGTTTTCT TATCTGCCCCAGCACTC			

KF6-F3	ACCATGCTGCGATTCATG	Junction of KF6	187	This work
KF6-B3	ATCTTCATCCCTGGACTTG			
KF6-FIP	GAGTGACACGAATTCAACCTGATTTTTG CACTAAATCAATACCTCCT			
KF6-BIP	GGCTTGCAAATCCTGCATGTTTTTGTAAC AGAAATCAGCAACGT			
